# Risk of ESRD and All Cause Mortality in Type 2 Diabetes According to Circulating Levels of FGF-23 and TNFR1

**DOI:** 10.1371/journal.pone.0058007

**Published:** 2013-03-20

**Authors:** Jung Eun Lee, Tomohito Gohda, William H. Walker, Jan Skupien, Adam M. Smiles, Rita R. Holak, Jackson Jeong, Kevin P. McDonnell, Andrzej S. Krolewski, Monika A. Niewczas

**Affiliations:** 1 Research Division, Joslin Diabetes Center, Boston, Massachusetts, United States of America; 2 Division of Nephrology, Samsung Medical Center, Sungkyunkwan University School of Medicine, Seoul, Korea; 3 Department of Medicine, Harvard Medical School, Boston, Massachusetts, United States of America; 4 Division of Nephrology, Department of Internal Medicine, Juntendo University School of Medicine, Tokyo, Japan; 5 Department of Metabolic Diseases, Jagiellonian University Medical College, Krakow, Poland; Children's Hospital Boston/Harvard Medical School, United States of America

## Abstract

**Introduction:**

Recent studies demonstrated that circulating fibroblast growth factor (FGF)-23 was associated with risk of end stage renal disease (ESRD) and mortality. This study aims to examine whether the predictive effect of FGF-23 is independent from circulating levels of tumor necrosis factor receptor 1 (TNFR1), a strong predictor of ESRD in Type 2 diabetes (T2D).

**Methods:**

We studied 380 patients with T2D who were followed for 8–12 years and were used previously to examine the effect of TNFR1. Baseline plasma FGF-23 was measured by immunoassay.

**Results:**

During follow-up, 48 patients (13%) developed ESRD and 83 patients (22%) died without ESRD. In a univariate analysis, baseline circulating levels of FGF-23 and TNFR1 were significantly higher in subjects who subsequently developed ESRD or died without ESRD than in those who remained alive. In a Cox proportional hazard model, baseline concentration of FGF-23 was associated with increased risk of ESRD, however its effect was no longer significant after controlling for TNFR1 and other clinical characteristics (HR 1.3, p = 0.15). The strong effect of circulating level of TNFR1 on risk of ESRD was not changed by including circulating levels of FGF-23 (HR 8.7, p<0.001). In the Cox multivariate model, circulating levels of FGF-23 remained a significant independent predictor of all-cause mortality unrelated to ESRD (HR 1.5, p<0.001).

**Conclusions:**

We demonstrated that the effect of circulating levels of FGF-23 on the risk of ESRD is accounted for by circulating levels of TNFR1. We confirmed that circulating levels of FGF-23 have an independent effect on all-cause mortality in T2D.

## Introduction

Diabetic nephropathy is one of the most devastating complications of diabetes. It remains the leading cause of end-stage renal disease (ESRD), accounting for 44% of ESRD incident cases in the United States [1]. Type 2 diabetes (T2D) also increases risk of mortality [2]. Increased urinary excretion of albumin has long been considered a major determinant of diabetic nephropathy progression. However, its value as an accurate marker of the progression to ESRD was recently challenged [3]–[5]. Thus, new markers that will better identify diabetes patients with at risk of ESRD or mortality unrelated to ESRD are needed.

Recently, results from the Joslin Kidney Study demonstrated that among several inflammatory markers measured, increased concentrations of circulating Tumor Necrosis Factor Receptor (TNFR) 1 and TNFR2 emerged as very strong predictors of diabetic nephropathy progression to chronic kidney disease (CKD) stage 3 or ESRD [6], [7]. TNFR1 and TNFR2 are cell membrane-bound receptors involved in apoptosis, inflammation and immune response [8]. They are released into the extracellular space by the action of a cleavage enzyme or by exocytosis within exosome-like vesicles [9]. It remains unclear how circulating levels of TNFRs impact risk of renal function decline in diabetes [6], [7].

Fibroblast growth factor (FGF)-23 is an endocrine hormone secreted by bone cells [10]. The primary physiologic actions of FGF-23 levels are to induce phosphaturia by decreasing urinary reabsorption in proximal tubule, to reduce active vitamin D production and to inhibit PTH secretion [11], [12]. Recent epidemiologic studies have focused on the prognostic values of plasma FGF-23 levels and demonstrated that the circulating level of FGF-23 is strongly associated with higher risk of ESRD and death in subjects with CKD [13]–[17]. Also, circulating levels of FGF-23 are associated with serum levels of several inflammatory markers in non-diabetic subjects with CKD [18], [19], and with circulating levels of TNFR1 in diabetic patients [20].

This study aims to evaluate the effect of circulating levels of FGF-23 on risk of ESRD and mortality unrelated to ESRD in a prospective study of T2D subjects. The question of great importance is whether the effect of TNFR1 can account for the effect of FGF-23, or are these two effects independent.

## Materials and Methods

### Study patients

The Joslin Kidney Study in T2D patients was previously described [7]. Briefly, a random sample of Joslin Clinic patients with T2D was recruited into the Joslin Study between 1991 and 1995. Eligibility criteria included residence in Massachusetts, T2D diagnosed between ages 35 and 64 years, and age at examination 40 to 69 years. The study protocol and informed written consent procedures were approved by the Joslin Diabetes Center Institutional Review Board. Trained recruiters performed a physical examination that included standardized measurements of blood pressure and collected samples of urine and blood biochemical determinations (stored at −80°C). Questionnaires were supplemented with data from medical records and clinical laboratory database. Of the 600 patients selected, 509 were examined and enrolled into the study. Patients with evidence of nephropathy unrelated to diabetes and patients in CKD stage 5 [defined as an estimated glomerular filtration rate (eGFR) <15 ml/min per 1.73 m^2^ using the Modified Diet in Renal Disease formula] were excluded. This left 410 patients, with 85% defining themselves as Caucasian. Three hundred eighty patients with available plasma samples for FGF-23 measurements were included in this study.

### Assessment of albuminuria status and estimated GFR at baseline

We determined the albumin to creatinine ratio (ACR, mg/g Cr) using the urine sample obtained at the baseline examination. The ACR value was converted to an albumin excretion rate (AER) according to a previously published formula [21]. This AER was used in the univariate and multivariate analyses.

In addition to the baseline urine, we retrieved the results of urinalysis performed on these patients' urine during the preceding two-year interval from the Joslin Clinical computer database, and converted it to an AER as previously described [21]. We determined geometric mean AER for the preceding two-year interval to assign an albuminuria status: normoalbuminuria (AER<30 µg/min), microalbuminuria (AER 30–300 µg/min) and macroalbuminuria/proteinuria (>300 µg/min).

Plasma creatinine was measured in stored baseline samples at the University of Minnesota with the Roche enzymatic assay (Prod No. 11775685) on a Roche/Hitachi Mod P analyzer. eGFR was obtained from plasma concentrations of creatinine using the IDMS-traceable Modified Diet in Renal Disease formula [5]. These measurements were performed in 2009.

### Measurements of plasma markers

All plasma markers were measured in baseline specimens by immunoassays in 2009. Circulating TNFR1 levels were determined with ELISA (Cat# DRT100, R&D Systems, Minneapolis, MN) as previously described [7]. Plasma concentrations of C-terminal FGF-23 were determined with ELISA (Cat# 60-6100, Immutopics, San Clemente, CA). All measurements were performed according to the manufacturer's protocols.

### Ascertainment of outcomes

The US Renal Data System (USRDS) maintains a roster of US patients receiving renal replacement therapy that includes dates of dialysis and transplantation [22]. The National Death Index (NDI) is a comprehensive roster of deaths in the United States, and includes date and causes of death [23]. All patients were queried against rosters of the USRDS and the NDI covering all events up to the end of 2004, as formerly reported [7].

### Statistical Analysis

Analyses were performed in SAS software (SAS Institute, Cary, NC, version 9.2). Differences among the three outcome groups were tested using the chi-squared test for categorical variables, and ANOVA with post hoc Tukey's t-test for continuous variables. Bonferroni correction was applied for the number of group comparisons. Spearman rank correlation matrix was created to evaluate the relationships among clinical variables and plasma markers. AER and the levels of markers were transformed to their logarithms for statistical analysis. Incidence rates of ESRD and deaths were tested for trend across quartiles of marker distribution using SAS macro provided by the Mayo Clinic [24], [25]. To evaluate the independent effects of markers for the prediction of outcome, we applied Cox proportional hazard models. P<0.05 was considered significant.

## Results

### Baseline characteristics of the study subjects according to outcomes

At study entry, the mean eGFR of the study group was 92±31 mL/min per 1.73 m^2^ and 325 subjects (86%) had preserved renal function (eGFR ≥60 mL/min per 1.73 m^2^). One hundred ninety five subjects (51%) had normoalbuminuria, 114 (30%) had microalbuminuria and 71 (19%) had proteinuria.

At the end of follow-up, 249 of the 380 subjects (65%) remained alive. ESRD had developed in 48 (13%) patients. The remaining 83 patients (22%) died without ESRD. Baseline characteristics are summarized in Table 1 according to three outcomes: Alive, ESRD, and Deceased. Those categorized as ESRD or Deceased were older, had longer duration of diabetes, higher AER and lower eGFR than those who remained Alive. The three outcome groups did not differ significantly with regard to HbA1c.

**Table 1:** Baseline characteristics of subjects with T2D according to their outcome during 8–12 years of follow-up.

Baseline Characteristics	Outcome			P-value	
	Alive (n = 249)	ESRD (n = 48)	Deceased (n = 83)	Alive vs ESRD	Alive vs Deceased
**Clinical Characteristics**					
Male (%)	54.6	43.8	65.1	0.1672	0.0959
Age (yr)	54±10	60±7	60±7	3.8×10^−4^	7.9×10^−7^
Duration of Diabetes (yr)	12±8	18±6	16±8	8.5×10^−7^	5.9×10^−5^
HbA1c (%)	8.3±1.7	8.9±1.5	8.6±1.6	0.0607	0.506
AER (µg/min)	20 (12–68)	657 (359–1544)	77 (20–217)	<10^−28^	4.8×10^−7^
eGFR (mL/min per 1.73 m^2^)	100±27	60±27	90±30	<10^−28^	0.0093
**Plasma Markers**					
TNFR1 (pg/mL)	1188 (1006–1447)	2543 (2151–3771)	1597 (1171–2079)	<10^−28^	5.8×10^−12^
FGF-23 (RU/mL)	50 (36–75)	117 (65–238)	84 (53–133)	5.6×10^−8^	2.9×10^−6^

Data are mean ± SD, median (25th, 75th percentiles), or percentage. AER and plasma markers were transformed to base 10 logarithms for the statistical analyses. Bonferroni correction for a number of groups was applied.

Concentrations of two markers in baseline plasma are also summarized in Table 1. As we previously reported, the ESRD group showed higher baseline concentrations of TNFR1 compared with the Alive group [7]. The Deceased group had levels that, while elevated, were only half as high as the ESRD group. Differences in plasma concentrations of FGF-23 according to outcome groups mirrored the pattern of TNFR1. However, the differences were weaker in case of FGF-23. Interestingly, the plasma concentrations of FGF-23 and TNFR1 in the total study subjects were only moderately correlated (Spearman correlation coefficient = 0.49, p<0.001).

### Results of Follow-up Study

To further evaluate the effects of plasma markers on the occurrence of ESRD and all-cause mortality, we used prospective analysis. During 8–12 years of follow-up the cohort of 380 patients with T2D had 3585 person-years of observation; 48 patients developed ESRD (incidence rate; 13/1000 person-years) and 83 died due to causes unrelated to ESRD (mortality rate; 23/1000 person-years). Incidence rate of ESRD increased from 3 to 6, 10 and 46 per 1000 person-years according to increasing quartiles of baseline FGF-23 (p<0.0001 for trend). An even more dramatic increase was seen for incidence rate of ESRD (rates 0, 1, 3 and 72 per 1000 person-years, p<0.0001 for trend) according to quartiles of baseline TNFR1. Mortality rates increased from 10 to 18, 26 and 49 per 1000 person-years with increasing quartiles of baseline FGF-23 (p<0.0001 for trend). Mortality rate increase (rates 13, 14, 26 and 53 per 1000 person-years, p<0.0001 for trend) according to quartiles of baseline TNFR1 were very similar to that observed for quartiles of baseline FGF-23. More detailed data about incidence rates of ESRD and all cause mortality according to both quartiles of baseline concentrations of FGF-23 and TNFR1 are shown in Table 2 and in Table S1, respectively.

**Table 2:** Incidence rate of ESRD in subjects with T2D stratified by quartiles of FGF-23 and TNFR1.

	FGF-23 Q1	FGF-23 Q2	FGF-23 Q3	FGF-23 Q4	Total
TNFR1 Q1					
Incidence rate (/1000 person-year)	0	0	0	0	0
No of Events/No of person-years	0/439	0/396	0/203	0/45	0/1083
No of subjects	37	36	18	4	95
TNFR1 Q2					
Incidence rate (/1000 person-year)	0	0	0	8.7	1.0
No of Events/No of person-years	0/355	0/246	0/302	1/115	1/1019
No of subjects	31	23	29	13	96
TNFR1 Q3					
Incidence rate (/1000 person-year)	4.8	0	0	10.4	3.4
No of Events/No of person-years	1/208	0/220	0/259	2/193	3/880
No of subjects	22	22	27	23	94
TNFR1 Q4					
Incidence rate (/1000 person-year)	52.8	55.9	55.4	91.3	72.9
No of Events/No of person-years	2/38	6/107	9/163	27/296	44/603
No of subjects	5	14	21	55	95
Total					
Incidence rate (/1000 person-year)	2.9	6.2	9.7	46.3	13.4
No of Events/No of person-years	3/1039	6/970	9/927	30/648	48/3585
No of subjects	95	95	95	95	380

Quartile cut-off values were 1049, 1302, and 1812 pg/mL for TNFR1 and 42, 60, and 96 RU/mL for FGF-23, respectively.

### Risk of ESRD according to both plasma markers

**Figure 1:**
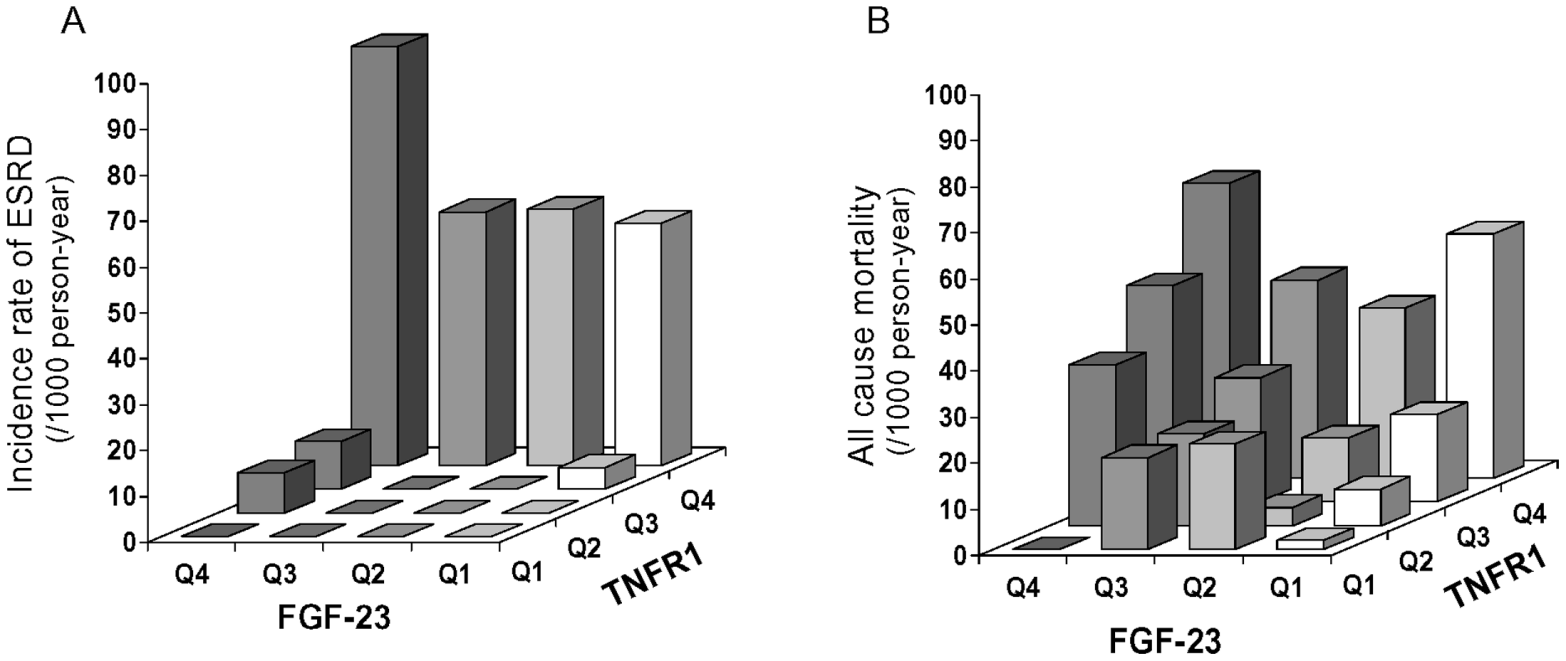
Incidence rate of ESRD and all-cause mortality stratified by quartiles of FGF-23 and TNFR1. Figure 1A demonstrates incidence rate of ESRD and Figure 1B shows incidence of all cause mortality. Q1–Q4 represents quartiles 1 to 4. Quartile cut-off values were 1049, 1302, and 1812 pg/mL for TNFR1 and 42, 60, and 96 RU/mL for FGF-23, respectively. Increasing color intensity of the columns corresponds to higher concentrations (quartiles) of FGF-23.

Incidence rates of ESRD by quartiles of baseline plasma concentrations of FGF-23 and TNFR1 are presented in Figure 1A. Darker bars represent higher concentrations of FGF-23. It was clear that although the rates increased with quartiles of FGF-23 in univariate analysis, the risk of ESRD was restricted almost exclusively to patients with the highest quartile of TNFR1. Among subjects with the highest quartile of TNFR1, the concentrations of FGF-23 did not discriminate the risk of ESRD (p = 0.13 for trend according to FGF-23 quartiles). Subjects who had plasma TNFR1 concentrations in quartiles 1-2 did not develop ESRD regardless of FGF-23 concentrations. To evaluate the effect of FGF-23 on the development of ESRD controlling for other clinical characteristics and plasma TNFR1, we used Cox proportional hazard models. The results are shown in Table 3. In a univariate analyses, clinical characteristics and both plasma markers were strongly associated with risk of ESRD. In multivariate analyses (model #1),when clinical characteristics were considered together with each marker, the hazard ratio (HR) for TNFR1 and FGF-23 declined significantly but still remained associated with risk of ESRD (HR 8.4, 95% C.I. 3.1–22.6 for one quartile increase of TNFR1 and HR 1.6, 95% C.I. 1.2–2.1 for one quartile increase of FGF-23). In model #2 when both markers were considered together with clinical characteristics, only the HR for TNFR1 was significant (HR 6.9, 95% C.I. 2.5–19.0 for one quartile increase). The effect of FGF-23 was not significant (HR 1.2, 95% C.I. 0.9–1.7 for one quartile increase).

**Table 3:** Univariate and multivariate Cox proportional hazard models assessing risk of ESRD adjusting for relevant baseline clinical characteristics and plasma markers in subjects with T2D followed for 8–12 years.

	Univariate analyses		Multivariate analyses			
			Model #1		Model #2	
	HR* (95% CI)	P-value	HR* (95% CI)	P-value	HR* (95% CI)	P-value
**Clinical Characteristics**						
AER	4.3 (3.2–5.9)	<0.0001	2.4 (1.7–3.5)	<0.0001	2.5 (1.7–3.6)	<0.0001
eGFR	2.0 (1.7–2.3)	<0.0001	1.3 (1.1–1.6)	0.0106	1.3 (1.1–1.6)	0.01
HbA1c	1.3 (1.0–1.6)	0.027	1.3 (1.1–1.7)	0.0092	1.4 (1.1–1.7)	0.008
**Plasma Markers**						
TNFR1	35.5 (15.0–84.0)	<0.0001	8.4 (3.1–22.6)	<0.0001	6.9 (2.5–19.0)	0.0002
FGF-23	2.8 (2.0–3.9)	<0.0001	1.6 (1.2–2.1)	0.002	1.2 (0.9–1.7)	0.15

* Effect measures are expressed as the HR for a one-quartile increase in the distribution of each covariate except for eGFR, for which it is a one-quartile decrease.

Model #1 included relevant clinical characteristics and plasma TNFR1 and FGF-23 independently.

Model #2 included relevant clinical characteristics and plasma TNFR1 and FGF-23 together.

### Mortality according to plasma markers

Rates of all-cause mortality according to quartiles of baseline FGF-23 and TNFR1 are presented in Figure 1B. Darker bars represent higher concentrations of FGF-23. Mortality rates clearly increased with quartiles of FGF-23 and with quartiles of TNFR1. The two effects were additive. To evaluate the effect of FGF-23 on the mortality controlling for other clinical characteristics and plasma TNFR1, we used Cox proportional hazard models. The results are shown in Table 4. In univariate analyses only two clinical characteristics, age and AER, were significant together with baseline concentrations of FGF-23 and TNFR1. In multivariate analyses (model #1) when clinical characteristics were considered together with each marker, the HRs for TNFR1 and FGF-23 declined somewhat but remained strongly associated with mortality (for one quartile increase of TNFR1, HR 1.4, 95% C.I. 1.1–1.7 and for one quartile increase of FGF-23, HR 1.6, 95% C.I. 1.3–2.0). In model #2 when both markers were considered together with clinical characteristics, the HR for FGF-23 was significant (for one quartile increase of TNFR1, HR 1.1, 95% C.I. 0.8–1.5 and for one quartile increase of FGF-23, HR 1.5, 95% C.I. 1.2–2.0).

**Table 4:** Univariate and multivariate Cox proportional hazard models assessing risk of all-cause mortality adjusting for relevant baseline clinical characteristics and plasma markers in subjects with T2D followed for 8–12 years.

	Univariate analyses		Multivariate analyses			
			Model #1		Model #2	
	HR* (95% CI)	P-value	HR* (95% CI)	P-value	HR* (95% CI)	P-value
**Clinical Characteristics**						
Age	1.6 (1.3–1.9)	<0.0001	1.4 (1.1–1.8)	<0.0001	1.4 (1.1–1.7)	0.0011
AER	1.4 (1.2–1.7)	<0.0001	1.2 (0.99–1.5)	0.059	1.3 (1.04–1.6)	0.0206
**Plasma Markers**						
TNFR1	1.9 (1.5–2.4)	<0.0001	1.4 (1.1–1.8)	0.012	1.1 (0.8–1.5)	0.42
FGF-23	1.8 (1.5–2.3)	<0.0001	1.6 (1.3–2.0)	<0.0001	1.5 (1.2–2.0)	0.0005

* Effect measures are expressed as the HR for a one-quartile increase in the distribution of each covariate except for eGFR, for which it is a one-quartile decrease.

Model #1 included relevant clinical characteristics and plasma TNFR1 and FGF-23 independently.

Model #2 included relevant clinical characteristics and plasma TNFR1 and FGF-23 together.

When cardiovascular death risk (n = 47) was analyzed separately, FGF-23 levels remained independent predictors in the model, which included age, AER and TNFR1 (effect for one quartile FGF-23 increase HR 1.4, 95% C.I. 1.0–2.0). More detailed results are presented in Table S2.

## Discussion

In our prospective study of subjects with T2D, we demonstrated that baseline plasma concentration of FGF-23 was associated with increased risk of ESRD. However its effect was no longer significant after controlling for plasma concentration of TNFR1. In other words, plasma concentration of TNFR1 accounted for the effect of FGF-23 on risk of ESRD. However, baseline level of FGF-23 was a significant independent predictor of all-cause as well as cardiovascular mortality unrelated to ESRD.

Recent epidemiologic studies reported association between plasma FGF-23 levels and clinical outcomes in patients with CKD [13]–[17]. Several cross-sectional studies demonstrated that FGF-23 levels were increased in patients with CKD [26]. Several reports show high levels of circulating FGF-23 as a predictor of progression to ESRD [13], [14], [17]. In the Chronic Renal Insufficiency Cohort Study during 3.5 years of follow-up elevated FGF-23 was an independent risk factor for ESRD [14]. In another follow-up study of 177 patients with non-diabetic CKD, higher levels of C-terminal FGF-23 and intact FGF-23 were independently associated with incident ESRD [17]. A small study of subjects with diabetes and impaired renal function at baseline reported that FGF-23 was a predictor of renal outcome independent of creatinine clearance, although its 12 ESRD events did not allow a fully adjusted Cox analysis [16].

The mechanisms are unclear as to which circulating FGF-23 may impact/be associated with impaired renal function and contributes to progression to ESRD. In non-diabetic subjects with impaired renal function, circulating levels of FGF-23 were correlated with serum concentrations of several markers of systemic inflammation such as IL-6, C-reactive protein and TNFα [18], [19]. One study reported that elevated FGF-23 levels were associated with TNFR1 levels in subjects with diabetic nephropathy [20]. Interestingly these findings were confirmed in our study. Table S3 shows correlations between baseline plasma levels of FGF-23 and ACR, eGFR and plasma markers such as CRP, IL-6, free and total TNFα, TNFR1 and TNFR2. Although these correlations were statistically significant, they were only moderate. The correlations between these markers and plasma level of TNFR1, a marker that accounted for the initial effect of FGF-23 on risk of ESRD in T2D, were almost twice as strong. These patterns of associations may indicate that both FGF-23 and TNFR1 (TNF markers) cause progression to ESRD in the same pathway. TNFR1 appeared to be stronger predictor, either because it is more directly involved in progression to ESRD or because its features as a biomarker are potentially better (i.e. better stability over time). Another possibility is that FGF-23 is simply a correlate of circulating level of TNFRs and is not causally related to progression to ESRD.

The role of FGF-23 on the inflammatory pathway has not yet been studied in depth. The effect of FGF-23 may be mediated via expression of Klotho. Klotho is an essential cofactor of FGF-23, expressed highly in renal tubules [27]. Higher FGF-23 levels may be associated with low Klotho tissue expression [28], [29]. Klotho expression is down-regulated in several kidney injury models and its over-expression attenuates renal damage in the experimental models of kidney injury [30]. Moreno et al. reported that TNF (TNFRs ligand) decreases Klotho expression [31]. The relation between expression of Klotho and plasma levels of TNFRs is unknown. On the other hand, exogenous administration of Klotho suppressed NF-kB activation and subsequent inflammatory cytokines production in in-vitro study [32]. A few studies examined the clinical implication of plasma Klotho levels in subjects with CKD, but failed to demonstrate consistent association of Klotho levels with renal function or poor outcome [33]. Additionally, increased FGF-23 levels reduce vitamin D activation, which has known anti- inflammatory properties [34], [35]. Increase of vitamin D levels by dietary supplement resulted in decrease of systemic inflammatory markers such as CRP and TNFα in subjects with T2D [36].

In contrast to the lack of independent effect of circulating FGF-23 on progression to ESRD, our study demonstrated that FGF-23 had an independent impact on risk of death unrelated to ESRD, including CVD deaths. Interestingly, in multivariate analyses, FGF-23 effect accounted for an effect of circulating TNFRs on mortality in T2D shown in our previous report [7]. The mechanism underlying the association between FGF-23 levels and mortality remains unclear. First, some investigators suggest that FGF-23 levels may be a sensitive surrogate marker for the toxicity of disturbance in phosphate and mineral metabolism in CKD patients [15]. However, the predictive effect of FGF-23 levels is not attenuated by serum phosphate, PTH, and vitamin D levels and FGF-23 levels are stronger predictors of mortality than other bone-related markers [13], [16]. Alternatively, FGF-23 levels may be a surrogate marker of the severity of CKD and subsequent increased risk of mortality. However, this scenario is also unlikely given the observation that the association with mortality was independent of TNFR1 levels in this study, while the association with ESRD was not. The third possibility is that elevated FGF-23 levels may be a causal factor contributing to increased mortality. This possibility is indirectly supported by the observation that higher FGF-23 levels are associated with vascular calcification, endothelial dysfunction and left ventricular hypertrophy in CKD patients [15], [37], [38].

Finally, we should mention a few limitations of our study. First, we measured only C-terminal, and not the intact form of FGF-23. However, a recent study showed the following: both forms are highly correlated; biologically active FGF-23 is accurately measured by either form; and clinical associations are comparably strong between the two [39]. Second, it is not clear how stable plasma concentration of FGF-23 is over a period of several years. For example, we showed that plasma concentrations of TNFR1 are very stable in patients with T1D over several years [6]. Third, our study was conducted in mostly Caucasian subjects with T2D so it is uncertain if our findings could be applied to Non-Caucasians and to the subjects with T1D.

## Supporting Information

Table S1 **All-cause mortality in subjects with T2D stratified by quartiles of FGF-23 and TNFR1.**
(DOC)

Table S2 **Univariate and multivariate Cox proportional hazard models assessing risk of cardiovascular mortality adjusting for relevant baseline clinical characteristics and plasma markers in subjects with T2D followed for 8–12 years.**
(DOC)

Table S3 **Spearman correlation coefficients among clinical variables and plasma markers at baseline.**
(DOC)
