# Supplementary material for: Risk of ESRD and All Cause Mortality in Type 2 Diabetes According to Circulating Levels of FGF-23 and TNFR1
Source: PLoS One. 2013 Mar 20;8(3):e58007. doi: 10.1371/journal.pone.0058007 (PMC3603950; doi:10.1371/journal.pone.0058007)
Supplement: Table S1 — All-cause mortality in subjects with T2D stratified by quartiles of FGF-23 and TNFR1. (DOC) [file pone.0058007.s001.doc]

Table S1. All-cause mortality in subjects with T2D stratified by quartiles of FGF-23 and TNFR1.

|  | FGF-23 Q1 | FGF-23 Q2 | FGF-23 Q3 | FGF-23 Q4 | Total |
| --- | --- | --- | --- | --- | --- |
| TNFR1 Q1  Incidence rate (/1000 person-year)  No of Events / No of person-years  No of subjects | 2.3  1 / 439  37 | 22.7  9 / 396  36 | 19.7  4 / 203  18 | 0  0 / 45  4 | 12.9  14 / 1083  95 |
| TNFR1 Q2  Incidence rate (/1000 person-year)  No of Events / No of person-years  No of subjects | 8.5  3 / 355  31 | 4.1  1 / 246  23 | 19.8  6 / 302  29 | 34.9  4 / 115  13 | 13.7  14 / 1019  96 |
| TNFR1 Q3  Incidence rate (/1000 person-year)  No of Events / No of person-years  No of subjects | 19.2  4 / 208  22 | 13.6  3 / 220  22 | 27.0  7 / 259  27 | 46.7  9 / 193  23 | 26.1  23 / 880  94 |
| TNFR1 Q4  Incidence rate (/1000 person-year)  No of Events / No of person-years  No of subjects | 52.8  2 / 38  5 | 37.3  4 / 107  14 | 43.1  7 / 163  21 | 64.3  19 / 296  55 | 53.0  32 / 603  95 |
| Total  Incidence rate (/1000 person-year)  No of Events / No of person-years  No of subjects | 9.6  10 / 1039  95 | 17.5  17 / 970  95 | 25.9  24 / 927  95 | 49.4  32 / 648  95 | 23.1  83 / 3585  380 |

Quartile cut-off values were 1049, 1302, and 1812 pg/mL for TNFR1 and 42, 60, and 96 RU/mL for FGF-23, respectively.
