# Supplementary material for: Risk of ESRD and All Cause Mortality in Type 2 Diabetes According to Circulating Levels of FGF-23 and TNFR1
Source: PLoS One. 2013 Mar 20;8(3):e58007. doi: 10.1371/journal.pone.0058007 (PMC3603950; doi:10.1371/journal.pone.0058007)
Supplement: Table S2 — Univariate and multivariate Cox proportional hazard models assessing risk of cardiovascular mortality adjusting for relevant baseline clinical characteristics and plasma markers in subjects with T2D followed for 8–12 years. (DOC) [file pone.0058007.s002.doc]

Table S2. Univariate and multivariate Cox proportional hazard models assessing risk of cardiovascular mortality adjusting for relevant baseline clinical characteristics and plasma markers in subjects with T2D followed for 8-12 years.

|  | Univariate analysis | | Multivariate analysis | | | |
| --- | --- | --- | --- | --- | --- | --- |
|  |  |  | Model #1 | | Model #2 | |
|  | HR* (95% CI) | P-value | HR* (95% CI) | P-value | HR*(95% CI) | P-value |
| **Clinical Characteristics** |  |  |  |  |  |  |
| Age | 1.5 (1.2-2.0) | 0.0005 | 1.3 (1.01-1.7) | 0.044 | 1.3 (1.002-1.7) | 0.049 |
| AER | 1.4 (1.1-1.8) | 0.0025 | 1.2 (0.9-1.5) | 0.265 | 1.2 (0.9-1.6) | 0.174 |
| **Plasma Marker~~r~~** |  |  |  |  |  |  |
| TNFR1 | 2.1 (1.6-2.8) | <0.0001 | 1.6 (1.1-2.4) | 0.008 | 1.4 (0.9-2.0) | 0.120 |
| FGF-23 | 1.8 (1.4-2.4) | <0.0001 | 1.6 (1.2-2.1) | 0.002 | 1.4 (1.04-2.0) | 0.028 |

*Effect measures are expressed as the HR for a one-quartile increase in the distribution of each covariate except for eGFR, for which it is a one-quartile decrease.

Model #1 included relevant clinical characteristics and plasma TNFR1 and FGF-23 independently.

Model #2 included relevant clinical characteristics and plasma TNFR1 and FGF-23 together.
