# Supplementary material for: Risk of ESRD and All Cause Mortality in Type 2 Diabetes According to Circulating Levels of FGF-23 and TNFR1
Source: PLoS One. 2013 Mar 20;8(3):e58007. doi: 10.1371/journal.pone.0058007 (PMC3603950; doi:10.1371/journal.pone.0058007)
Supplement: Table S3 — Spearman correlation coefficients among clinical variables and plasma markers at baseline. (DOC) [file pone.0058007.s003.doc]

Table S3. Spearman correlation coefficients among clinical variables and plasma markers at baseline.

|  | FGF-23 | TNFR1† |
| --- | --- | --- |
| Age | 0.31* | 0.48* |
| HbA1c | 0.08 | 0.08 |
| ACR | 0.23* | 0.51* |
| eGFR | -0.33* | -0.59* |
| CRP† | 0.30* | 0.29* |
| IL-6† | 0.39* | 0.50* |
| VCAM-1† | 0.10 | 0.32 |
| ICAM-1† | 0.17 | 0.30 |
| Free TNFα† | 0.27* | 0.50* |
| Total TNFα† | 0.42* | 0.76* |
| TNFR1† | 0.49* | 1.0 |
| TNFR2† | 0.47* | 0.90* |

*p<0.0001

†These measurements were adopted from the previous paper on Joslin Kidney Study in T2D patients [7].
